# Supplementary material for: Analysis of genome and methylation changes in Chinese indigenous chickens over time provides insight into species conservation
Source: Commun Biol. 2022 Sep 12;5:952. doi: 10.1038/s42003-022-03907-7 (PMC9467985; doi:10.1038/s42003-022-03907-7)
Supplement: Supplementary file 2 — Supplementary Information [file 42003_2022_3907_MOESM2_ESM.pdf]

Supplementary Information

Analysis of genome and methylation changes in Chinese indigenous chickens over time provides insight into species conservation

Zeng, et al.

This PDF file includes:

Supplementary Figure 1-10

Supplementary Table 1-3

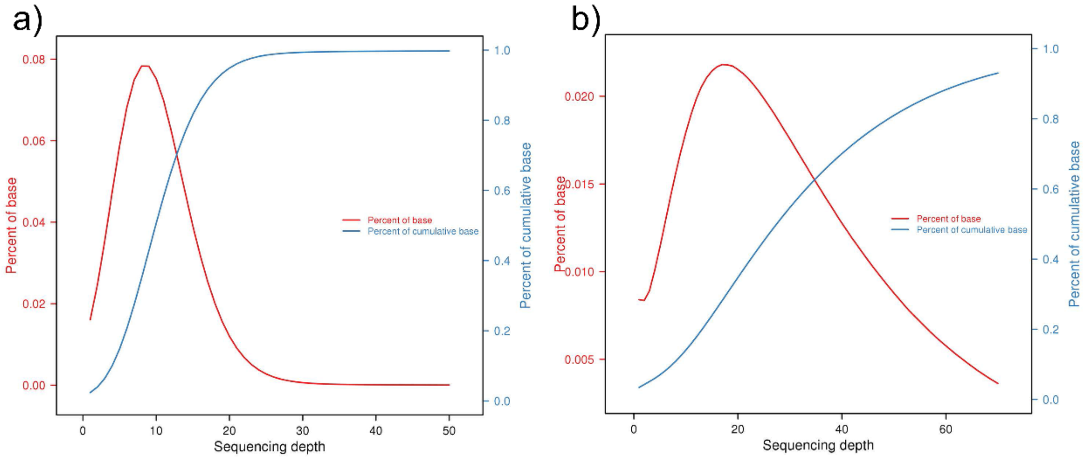

Supplementary Figure 1. The basic distribution of sequencing depth. The abscissa represents the sequencing depth, the red curve represents the percentage of bases corresponding to that depth, and the blue curve represents the percentage of bases corresponding to that depth and below. **a)** Whole-genome resequencing. **b)** Whole-genome bisulfite sequencing.

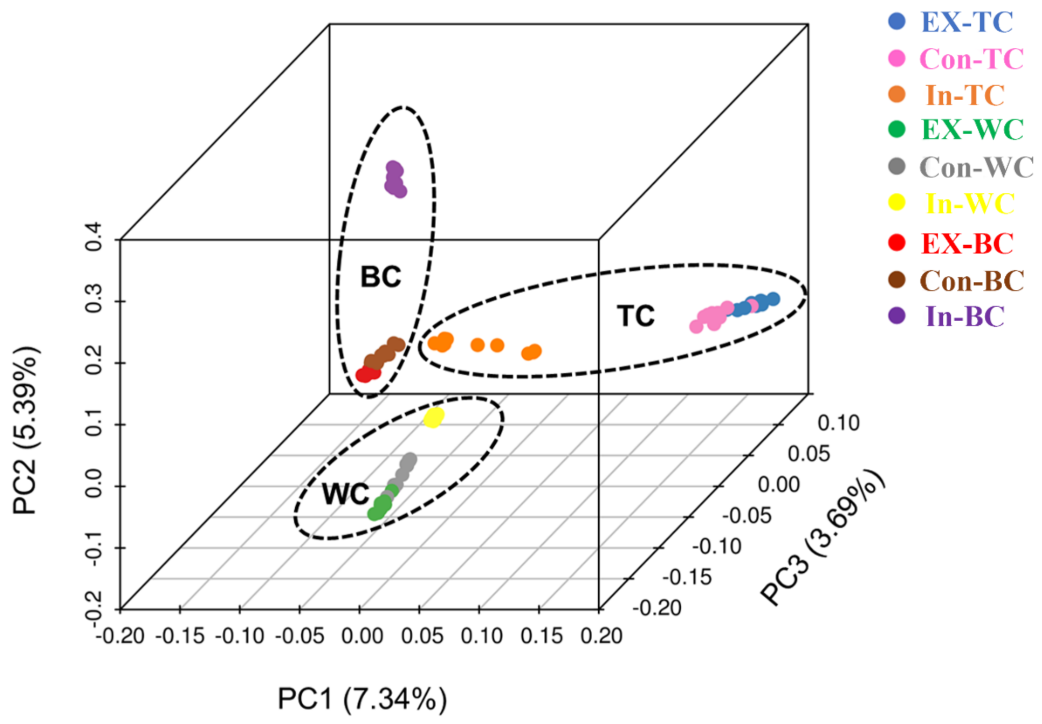

Supplementary Figure 2. The principal component analysis (PCA) of three-dimensional cluster diagram for 90 indigenous chickens (TC: Tibetan chicken, WC: Wenchang chicken and BC: Bian chicken). Each breed was divided into three groups according to the conservation programmes (Con – cryopreserved samples, as a control, NCF; In – *in situ* conservation, NCF; and Ex – *ex situ in vivo* conservation, NCGR).

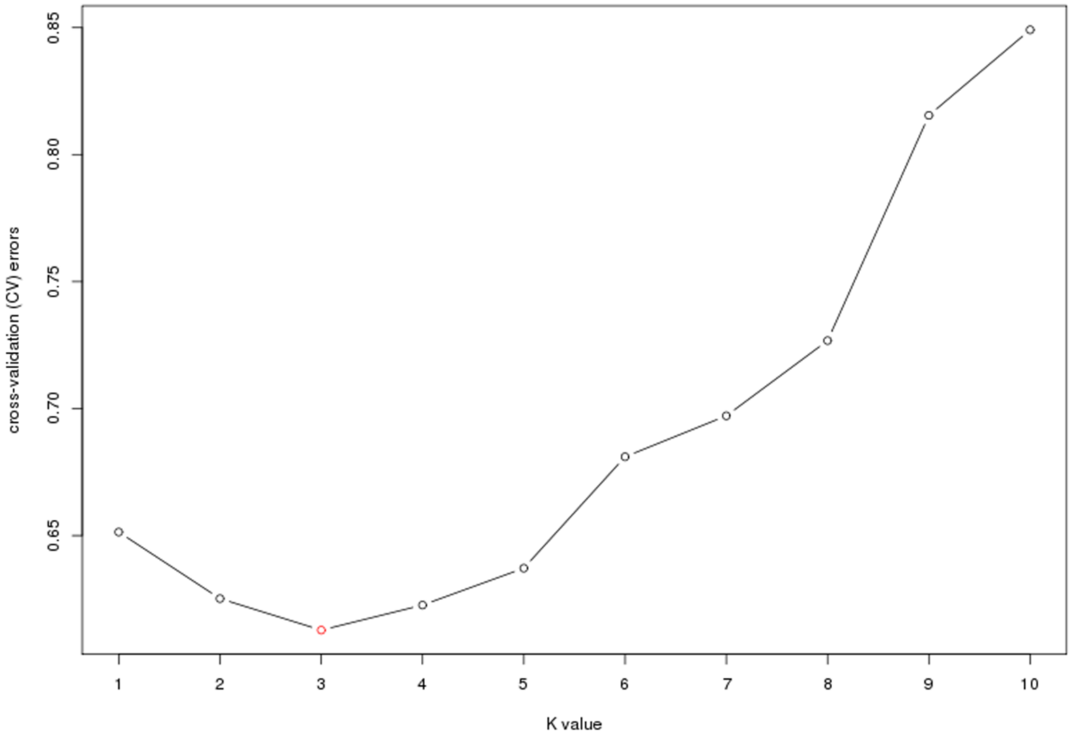

Supplementary Figure 3. ADMIXTURE cross validation errors for each ancestral cluster value (K) of 9 populations from 3 indigenous chicken breeds.

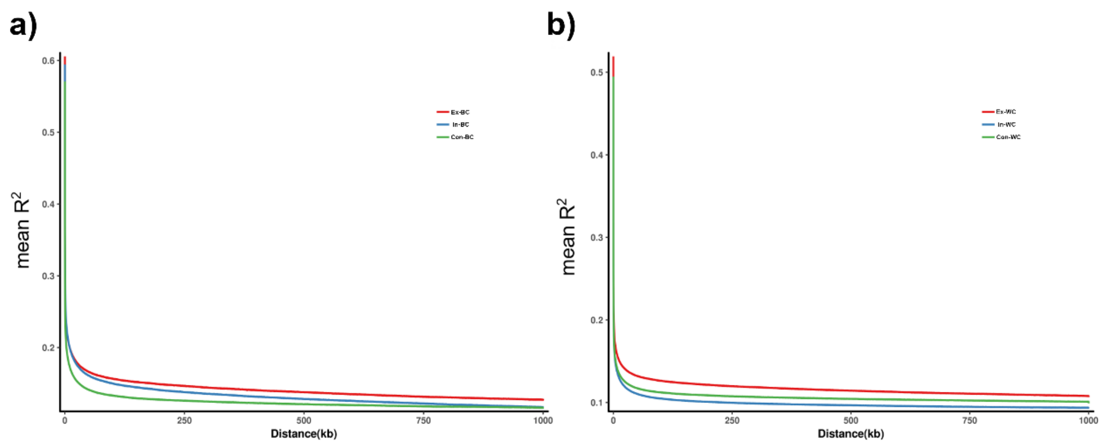

Supplementary Figure 4. **Patterns of LD (linkage disequilibrium) decay across the genome during different conservation programmes (Con – cryopreserved samples, as a control, NCF; In – *in situ* conservation, NCF; and Ex – *ex situ in vivo* conservation, NCGR).** X axis: physical distances between two SNPs marked in kb; Y axis: R^2, pearson's correlation coefficient, used to measure LD. **a)** LD decay in Wenchang chicken (WC). **b)** LD decay in Bian chicken (BC).

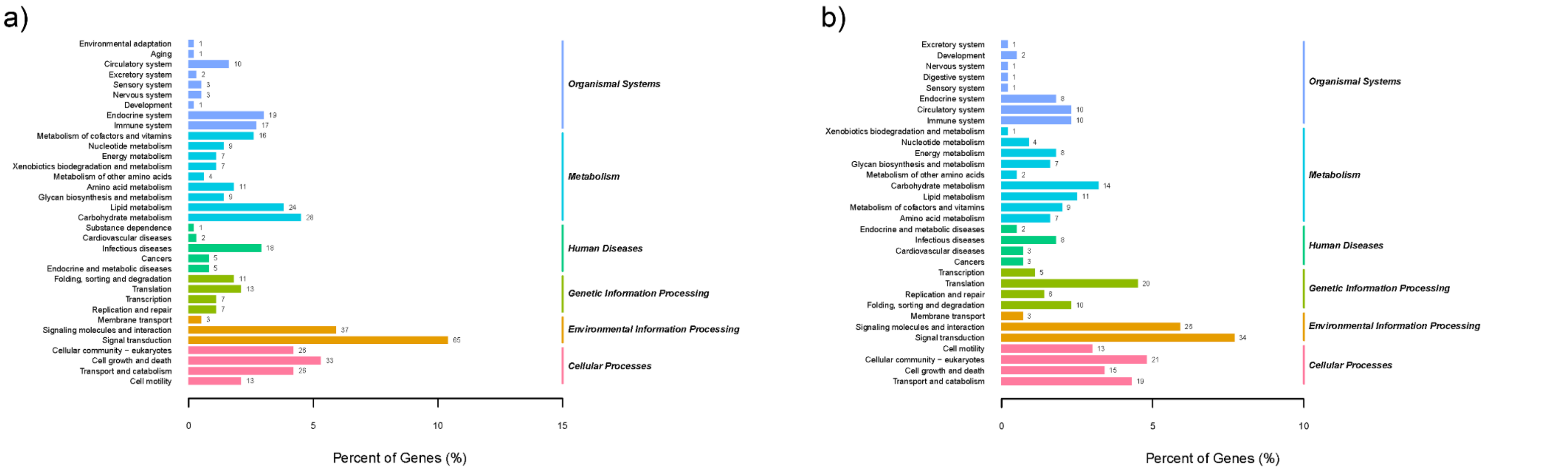

Supplementary Figure 5. **Kyoto Encyclopedia of Genes and Genomes (KEGG) pathway analysis on the selected genes between Con-TC and other chickens (Con-BC and Con-WC). a)** KEGG pathway analysis on the selected genes between Con-TC and Con-BC. **b)** KEGG pathway analysis on the selected genes between Con-TC and Con-WC.

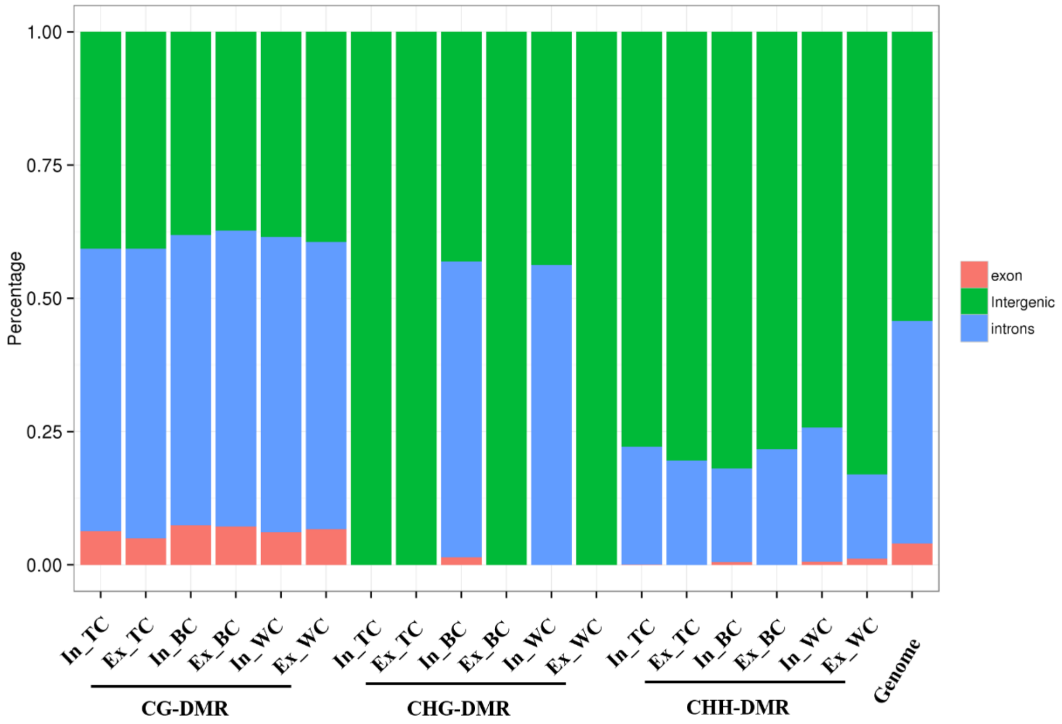

Supplementary Figure 6. Genomic compositions of different DMR types (CG, CHG and CHH) during *in situ* (In) and *ex situ in vivo* (Ex) conservation programmes of chickens (TC: Tibetan chicken, WC: Wenchang chicken and BC: Bian chicken).

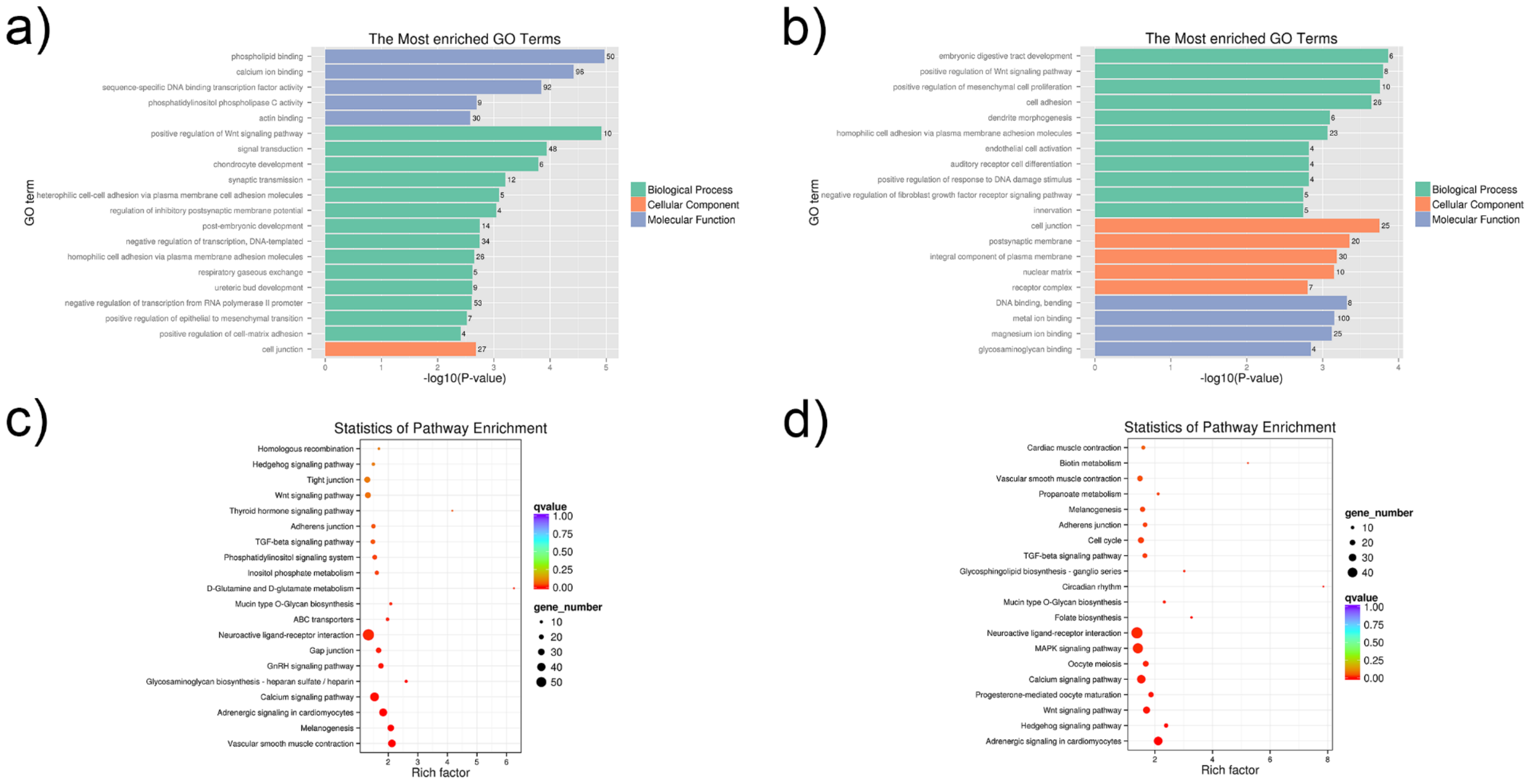

Supplementary Figure 7. Top GO and top KEGG pathway analysis of CG type DMGs during TC conservation. a, c) Top GO analysis and top KEGG analysis during *ex situ in vivo* conservation of TC (Ex_TC). **b, d)** Top GO analysis and top KEGG analysis during *in situ* conservation of TC (In_TC).

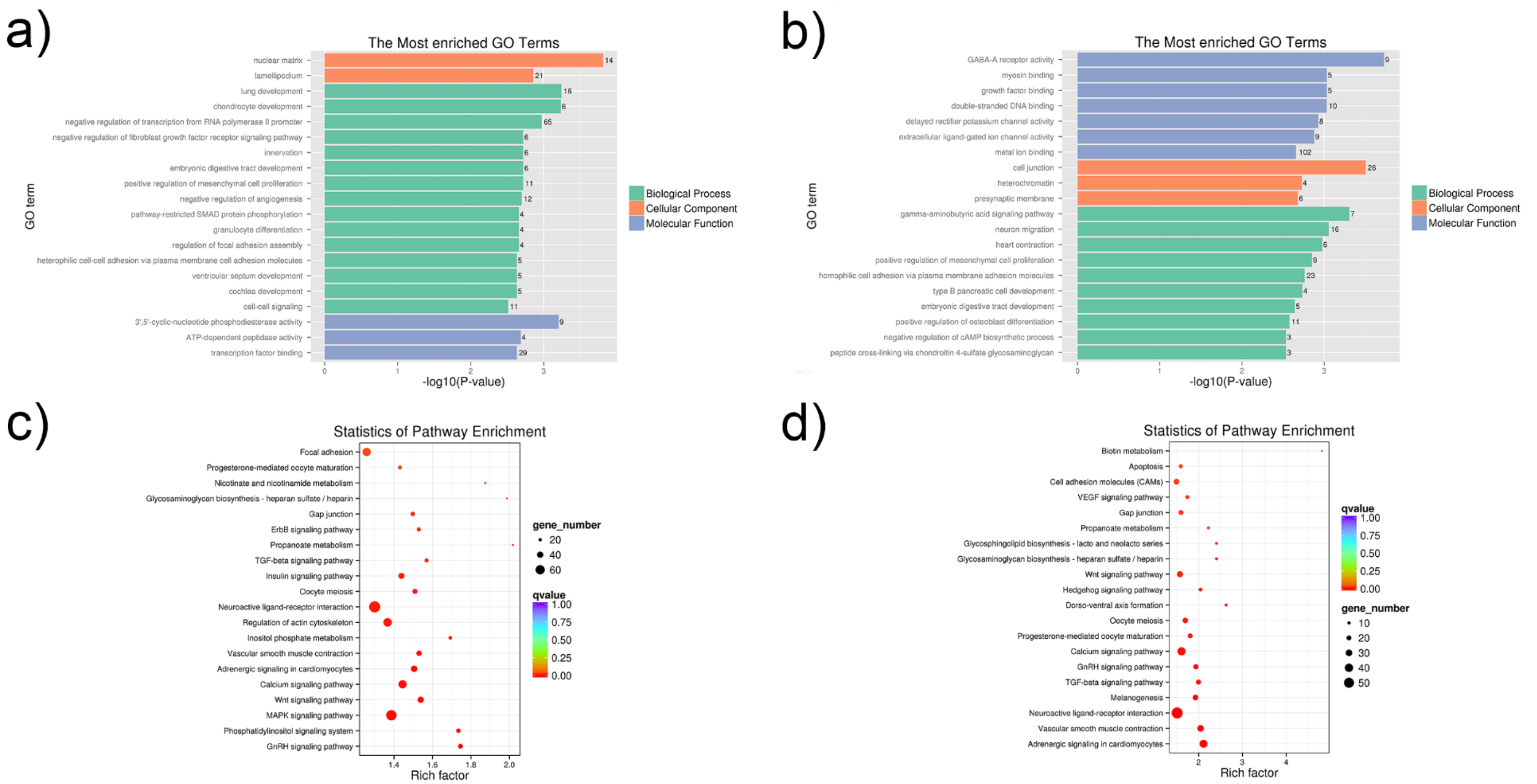

Supplementary Figure 8. **Top GO and top KEGG pathway analysis of CG type DMGs during WC conservation. a, c)** Top GO analysis and top KEGG analysis during *ex situ in vivo* conservation of WC (Ex_WC). **b, d)** Top GO analysis and top KEGG analysis during *in situ* conservation of WC (In_WC).

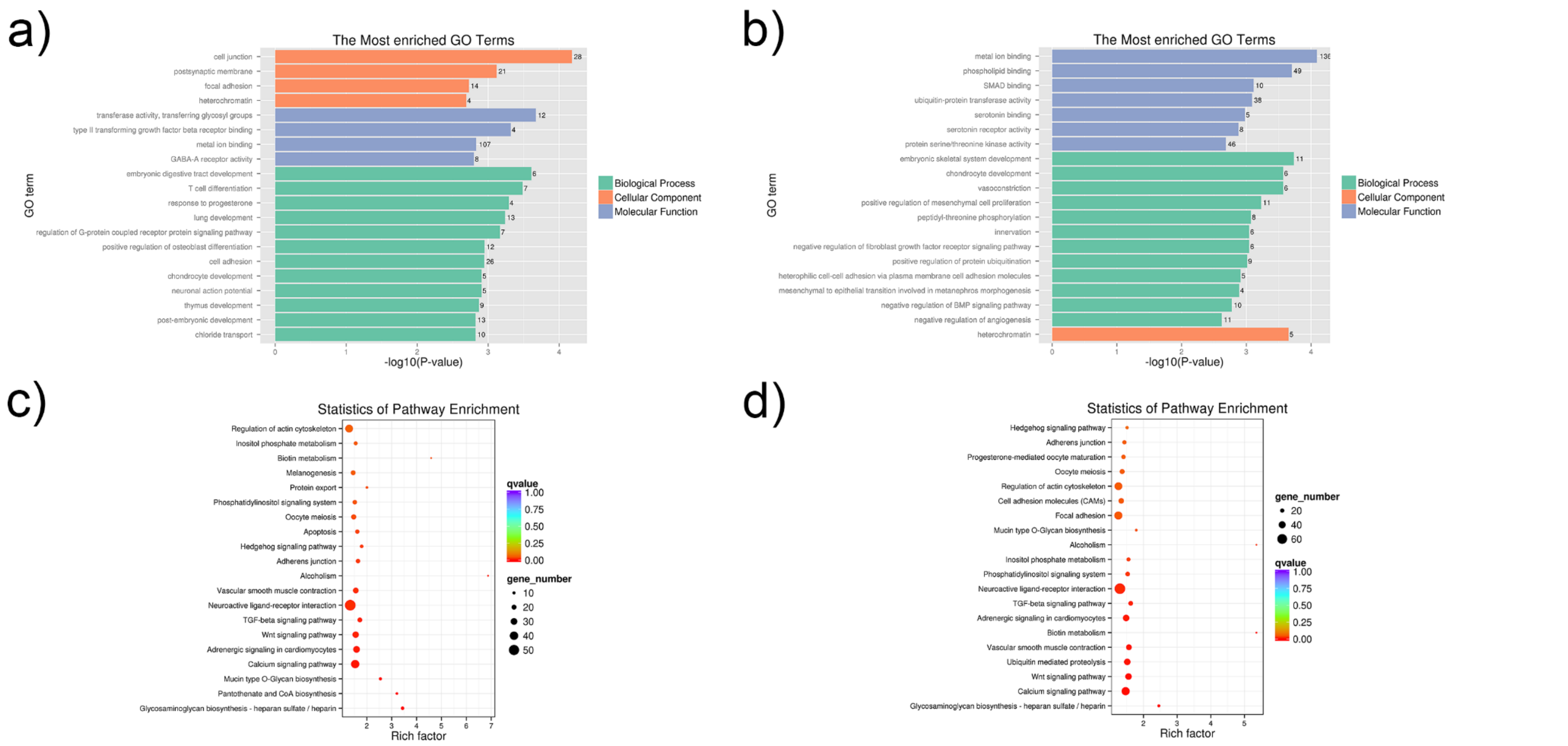

Supplementary Figure 9. Top GO and top KEGG pathway analysis of CG type DMGs during BC conservation. a, c) Top GO analysis and top KEGG analysis during *ex situ in vivo* conservation of BC (Ex_BC). **b, d)** Top GO analysis and top KEGG analysis during *in situ* conservation of BC (In_BC).

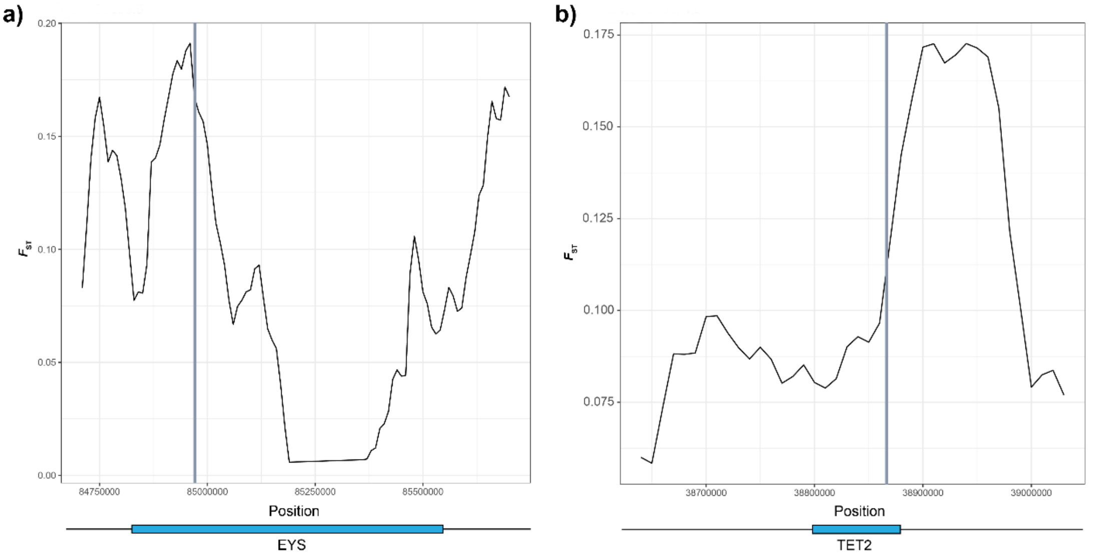

Supplementary Figure 10. **Example of two genes named *EYS* (a) and *TET2* (b), which located in s-DMRs during *ex situ in vivo* conservation programme of Tibetan chickens.** Gray shadow depicts the location of DMRs.

Supplementary Table 1. Information of chicken breeds used in this study.

Breed	Geographic Origin	Risk Status	Abbreviation	Conservation Actions	Conservation Location	Altitudes (m)	Temperature zones	No. Sample	Sampling Date (Year)
Tibetan Chicken	Lhasa, Tibet	Not at risk	Con-TC	/	Lhasa, Tibet	3,700	Qinghai–Tibetan Plateau	10	2000
			In-TC	*in situ*	Lhasa, Tibet	3,700	Qinghai–Tibetan Plateau	10	2019
			Ex-TC	*ex situ* in vivo	Yangzhou, Jiangsu	⩽20	Subtropical	10	2019
Wenchang Chicken	Wenchang, Hainan	Not at risk	Con-WC	/	Wenchang, Hainan	⩽20	Tropical	10	2000
			In-WC	*in situ*	Wenchang, Hainan	⩽20	Tropical	10	2019
			Ex-WC	*ex situ* in vivo	Yangzhou, Jiangsu	⩽20	Subtropical	10	2019
Bian Chicken	Shouzhou, Shanxi	Endangered	Con-BC	/	Shouzhou, Shanxi	1,400	temperate	10	2000
			In-BC	*in situ*	Shouzhou, Shanxi	1,400	temperate	10	2019
			Ex-BC	*ex situ* in vivo	Yangzhou, Jiangsu	⩽20	Subtropical	10	2019

Supplementary Table 2. A summary of WGBS data.

Breed	Abbreviation	Clean_Reads	Q30(%)	Mapped(%)	Ave_depth	Cov_ratio_1X(%)	Cov_ratio_5X(%)	Cov_ratio_10X(%)
Tibetan Chicken	Con-TC	132,148,768	92.77	71.72	24	96.51	92.64	84.42
	In-TC	113,579,478	90.96	68.75	19	96.14	89.74	76.58
	Ex-TC	159,539,250	90.17	70.63	28	96.58	93.01	85.94
Wenchang Chicken	Con-WC	132,306,135	93.20	75.67	25	96.36	92.29	84.16
	In-WC	120,099,861	90.42	67.12	17	96.00	88.04	72.42
	Ex-WC	145,966,026	93.11	73.69	27	96.48	92.60	85.13
Bian Chicken	Con-BC	130,625,642	92.70	69.28	22	96.16	90.93	80.20
	In-BC	122,479,187	91.11	70.71	21	96.33	91.54	81.21
	Ex-BC	133,935,485	92.91	72.07	24	96.51	92.66	84.53

Supplementary Table 3. Genetic diversity assessment for nine sub-populations of three chicken breeds.

Breed	Abbreviation	P_N (%)	*F*	*Ne*	θw	Tajima's D
Tibetan Chicken	Con-TC	56.85	0.1342	337.90	1.94E-03	0.9398
	In-TC	68.55	0.1466	396.20	2.16E-03	0.8228
	Ex-TC	52.64	0.1283	313.70	1.83E-03	1.0122
Wenchang Chicken	Con-WC	60.94	0.1465	161.70	2.05E-03	0.8639
	In-WC	65.06	0.1122	160.40	2.10E-03	0.8369
	Ex-WC	56.87	0.1404	167.60	1.95E-03	0.9401
Bian Chicken	Con-BC	52.06	0.1407	72.90	1.86E-03	0.8892
	In-BC	49.24	0.1036	192.70	1.77E-03	0.9878
	Ex-BC	46.76	0.1334	122.80	1.70E-03	1.0138

Note: P_N, proportion of polymorphic markers; *F*, inbreeding coefficient; *Ne*, effective population size; θw, Watterson's theta.
