# Supplementary material for: Analysis of genome and methylation changes in Chinese indigenous chickens over time provides insight into species conservation
Source: Commun Biol. 2022 Sep 12;5:952. doi: 10.1038/s42003-022-03907-7 (PMC9467985; doi:10.1038/s42003-022-03907-7)
Supplement: Supplementary file 3 — Description of Additional Supplementary Files [file 42003_2022_3907_MOESM3_ESM.docx]

**Description of Additional Supplementary Files**

**File name:** Supplementary Data 1
**Description:** Overview of whole genome sequencing data.

**File name:** Supplementary Data 2
**Description:** SNP and InDel summary statistics in the chicken breeds.

**File name:** Supplementary Data 3
**Description:** Methylated cytosine site statistics for each sequenced accession.

**File name:** Supplementary Data 4
**Description:** Candidate divergent regions (CDRs) and genes between Con-TC and Con-BC with the windows by top 5% highest (FST).

**File name:** Supplementary Data 5
**Description:** Candidate divergent regions (CDRs) and genes between Con-TC and Con-WC with the windows by top 5% highest (FST).

**File name:** Supplementary Data 6
**Description:** GO enrichment analysis on the selected genes between ConTC and Con-BC.

**File name:** Supplementary Data 7
**Description:** GO enrichment analysis on the selected genes between ConTC and Con-WC.

**File name:** Supplementary Data 8
**Description:** The same GO terms for genes showing high population differentiation between Con-TC and other chickens (Con-BC and Con-WC).

**File name:** Supplementary Data 9
**Description:** List of detected DMRs in Ex_TC.

**File name:** Supplementary Data 10
**Description:** List of detected DMRs in In_TC.

**File name:** Supplementary Data 11
**Description:** List of detected DMRs in Ex_WC.

**File name:** Supplementary Data 12
**Description:** List of detected DMRs in In_WC.

**File name:** Supplementary Data 13
**Description:** List of detected DMRs in Ex_BC.

**File name:** Supplementary Data 14
**Description:** List of detected DMRs in In_BC.

**File name:** Supplementary Data 15
**Description:** List of locally associated regions between DMRs and genomic selection signatures in Ex_TC (sweep DMRs).

**File name:** Supplementary Data 16
**Description:** Summary of candidate genes that may have a role in Ex_TC.

**File name:** Supplementary Data 17
**Description:** Numerical source data for Figures.

**File name:** Supplementary Software 1.
**Description:** Rcode.
