# Supplementary material for: Analysis of genome and methylation changes in Chinese indigenous chickens over time provides insight into species conservation
Source: Commun Biol. 2022 Sep 12;5:952. doi: 10.1038/s42003-022-03907-7 (PMC9467985; doi:10.1038/s42003-022-03907-7)
Supplement: Supplementary file 22 — Reporting Summary [file 42003_2022_3907_MOESM22_ESM.pdf]

nature research

Corresponding author(s): Wei Han and Lizhi Lu
Last updated by author(s): Aug 26, 2022

Reporting Summary

Nature Research wishes to improve the reproducibility of the work that we publish. This form provides structure for consistency and transparency in reporting. For further information on Nature Research policies, see our Editorial Policies and the Editorial Policy Checklist.

Statistics

For all statistical analyses, confirm that the following items are present in the figure legend, table legend, main text, or Methods section.

n/a	Confirmed	
☐	☒	The exact sample size (*n*) for each experimental group/condition, given as a discrete number and unit of measurement
☒	☐	A statement on whether measurements were taken from distinct samples or whether the same sample was measured repeatedly
☐	☒	The statistical test(s) used AND whether they are one- or two-sided<br>*Only common tests should be described solely by name; describe more complex techniques in the Methods section.*
☒	☐	A description of all covariates tested
☐	☒	A description of any assumptions or corrections, such as tests of normality and adjustment for multiple comparisons
☐	☒	A full description of the statistical parameters including central tendency (e.g. means) or other basic estimates (e.g. regression coefficient) AND variation (e.g. standard deviation) or associated estimates of uncertainty (e.g. confidence intervals)
☐	☒	For null hypothesis testing, the test statistic (e.g. F, t, r) with confidence intervals, effect sizes, degrees of freedom and P value noted<br>*Give P values as exact values whenever suitable.*
☐	☒	For Bayesian analysis, information on the choice of priors and Markov chain Monte Carlo settings
☐	☒	For hierarchical and complex designs, identification of the appropriate level for tests and full reporting of outcomes
☐	☒	Estimates of effect sizes (e.g. Cohen's *d*, Pearson's *r*), indicating how they were calculated

Our web collection on statistics for biologists contains articles on many of the points above.

Software and code

Policy information about availability of computer code

Data collection	Three Chinese indigenous chicken breeds that have been conserved in different conservation programmes (in situ and ex situ in vivo) were used in this study (TC: Tibetan chicken; WC: Wenchang chicken; and BC: Bian chicken). Each breed was divided into the following three groups according to the conservation programmes (Con – cryopreserved samples, as a control, in March 2000, kept in the NCF; In – in situ conservation after approximately 20 years, in March 2019, kept in the NCF; and Ex – ex situ in vivo conservation after approximately 20 years, in March 2019, kept in the NCGR) (Fig. 1a and Supplementary Table 1). Blood samples were collected from a total of 90 individuals, comprising 30 for each breed and 10 for each group in the same breed. Genome DNA was extracted using a DNA isolation kit (Tiangen, Beijing, China) according to the manufacturer's instructions. For genome sequencing, a minimum of 0.5 μg of genomic DNA from each sample was used to construct a library with an insert size of ~ 350 bp. Paired-end (PE) sequencing libraries were constructed according to the manufacturer's instructions (Illumina Inc., San Diego, CA, USA) and sequenced on an Illumina HiSeq 4000 platform (Illumina, San Diego, CA, USA). WGBS libraries were prepared according to the protocol described in a previous report74. The DNA samples were fragmented with sonication and subjected to bisulfite conversion. Ultra-high-throughput paired-end sequencing was carried out using the Illumina HiSeq 4000 platform (Illumina, San Diego, CA, USA) according to the manufacturer instructions. Raw HiSeq sequencing data were processed by Illumina base-calling pipeline (SolexaPipeline-1.0).
Data analysis	The list of Software used in this study are as follows:<br>Burrows Wheeler Aligner (v0.7.8)<br>SAMtools (v1.2)<br>GATK (v3.7)<br>SnpEff software (v4.3)<br>MEGA X software (v10.1.5)<br>ADMIXTURE (v1.3.0)<br>EIGENSOFT software (v7.2.1)<br>PLINK (v1.07)<br>NeESTIMATOR software (v1.3)

VCFtools (v0.1.14)
Haploview (v4.2)
Bismark (v0.21.0)
MOABS (v1.3.7.7)
Blast2GO (v5.2.5)

For manuscripts utilizing custom algorithms or software that are central to the research but not yet described in published literature, software must be made available to editors and reviewers. We strongly encourage code deposition in a community repository (e.g. GitHub). See the Nature Research guidelines for submitting code & software for further information.

Data

Policy information about availability of data

All manuscripts must include a data availability statement. This statement should provide the following information, where applicable:

- Accession codes, unique identifiers, or web links for publicly available datasets
- A list of figures that have associated raw data
- A description of any restrictions on data availability

All raw sequencing reads have been deposited in the National Center for Biotechnology Information (NCBI) Sequence Read Archive database (https://www.ncbi.nlm.nih.gov/sra) under project PRJNA698651 and PRJNA699090.

Field-specific reporting

Please select the one below that is the best fit for your research. If you are not sure, read the appropriate sections before making your selection.

☒ Life sciences ☐ Behavioural & social sciences ☐ Ecological, evolutionary & environmental sciences

For a reference copy of the document with all sections, see nature.com/documents/nr-reporting-summary-flat.pdf

Life sciences study design

All studies must disclose on these points even when the disclosure is negative.

Sample size	Blood samples were collected from a total of 90 individuals, comprising 30 for each breed and 10 for each group in the same breed.
Data exclusions	No data was excluded as the quality of whole-genome and whole-genome bisulfite sequencing data was good enough for all samples.
Replication	Blood samples were collected from a total of 90 individuals, comprising 30 for each breed and 10 for each group in the same breed.
Randomization	Genotypes were fully random in each different rearing batch. Genotypes were not known prior to methylation analyses, therefore the study was randomized.
Blinding	The aim was to investigate the genetic and DNA methylation variations occurring during different conservation programmes of Chinese indigenous chicken resources and to identify candidate genomic regions and genes underlying high-altitude adaptation in TC. The samples were grouped according to the source and conservation programmes of the sample. So blind experiment is not required for our work.

Reporting for specific materials, systems and methods

We require information from authors about some types of materials, experimental systems and methods used in many studies. Here, indicate whether each material, system or method listed is relevant to your study. If you are not sure if a list item applies to your research, read the appropriate section before selecting a response.

Materials & experimental systems

n/a	Involved in the study	
☒	☐	Antibodies
☒	☐	Eukaryotic cell lines
☒	☐	Palaeontology and archaeology
☐	☒	Animals and other organisms
☒	☐	Human research participants
☒	☐	Clinical data
☒	☐	Dual use research of concern

Methods

n/a	Involved in the study	
☒	☐	ChIP-seq
☒	☐	Flow cytometry
☒	☐	MRI-based neuroimaging

Animals and other organisms

Policy information about studies involving animals; ARRIVE guidelines recommended for reporting animal research

Laboratory animals	Chicken (Gallus gallus), reared to adults, males and females used.
Wild animals	The study did not involve wild animals.
Field-collected samples	The study did not involve samples collected from the field
Ethics oversight	Animals used in this study were raised in accordance with the national standard of Laboratory Animal Guidelines for ethical review of animal welfare. All experiment procedures were approved by the Zhejiang Academy of Agricultural Sciences (Hangzhou, China).

Note that full information on the approval of the study protocol must also be provided in the manuscript.
